# Influence of Melatonin on the Immune System of Fish: A Review

**DOI:** 10.3390/ijms14047979

**Published:** 2013-04-11

**Authors:** M. Ángeles Esteban, Alberto Cuesta, Elena Chaves-Pozo, José Meseguer

**Affiliations:** 1 Fish Innate Immune System Group, Department of Cell Biology and Histology, Faculty of Biology, International Excellence Campus, “Campus Mare Nostrum”, University of Murcia, 30100 Murcia, Spain; E-Mails: alcuesta@um.es (A.C.); meseguer@um.es (J.M.); 2 Marine Culture Plant of Mazarrón, Spanish Institute of Oceanography (IEO), Azohía Street, Puerto de Mazarrón, 30860 Murcia, Spain; E-Mail: elena.chaves@mu.ieo.es

**Keywords:** endocrine-immune system, rhythms, season, melatonin, immunity, fish

## Abstract

Endocrine-immune system interactions have been widely demonstrated in mammals, whereas in fish, these relationships remain unclear. Of the organs that constitute the endocrine system, the pineal gland and its secretory product melatonin act in the synchronization of daily and seasonal rhythms in most vertebrates, including fish. Seasonal differences in immunocompetence and disease prevalence have been well documented in humans. Seasonality also strongly influences the life history of fish by controlling the timing of physiological events, such as reproduction, food intake, locomotor activity, and growth performance. Apart from its synchronizing capabilities, the role of melatonin in physiological processes in fish is not thoroughly understood. The purpose of this review is to summarize current studies on the effects of melatonin on the fish immune system. These studies suggest that melatonin represents an important component of fish endocrine-immune system interactions. The elucidation of the defense mechanisms of fish will facilitate the development of health management tools to support the growing finfish aquaculture industry as well as address questions concerning the origins and evolution of the immune system in vertebrates.

## 1. Introduction

According to the Food and Agriculture Organization of the United Nations (FAO), fisheries and aquaculture make crucial contributions to worldwide prosperity. While the rate of captures by fisheries remains relatively stable, aquaculture production continues to expand and will likely remain one of the fastest-growing animal food-producing sectors. In the next decade, total production from fisheries and aquaculture will exceed that of beef, pork, or poultry. In 2010, the global production of farmed fish was 59.9 million tonnes, which represents an increase of 7.5% from the 55.7 million tonnes produced in 2009 [1].

The success of modern aquaculture is based on technological innovation and improved knowledge of the biology of farmed fish, which have permitted reproductive control and the development of specific feeds for each fish species. However, in large-scale production facilities, aquatic animals are exposed to stressful conditions, and the prevention of disease and environmental deterioration, which often result in economic losses, remains challenging [2]. The elucidation of fish defense mechanisms would facilitate the development of health management tools to support a growing finfish aquaculture industry and the improvement of present aquaculture practices. In addition, fish are considered an important model in studies of comparative immunology because they are a representative population of lower vertebrates that serve as an essential link to early vertebrate evolution [3].

A large body of evidence suggests that the nervous and endocrine systems interact with the immune system in vertebrates. Many neuroendocrine responses exhibit circadian and circannual rhythmicity, and given the close relationship between these systems, some immune parameters also exhibit both types of rhythmicity. The influence of melatonin on the immune system has been supported by several findings in birds and mammals: (a) a correlation has been demonstrated between melatonin production and circadian and seasonal variations in immune function; (b) pinealectomy has been shown to result in changes in the immune system; (c) *in vivo* and *in vitro* administration of melatonin has been shown to result in changes in immune functions; and (d) leucocyte receptors have been shown to be responsive to melatonin (see [4]). These observations are based on data obtained from birds and mammals, although other vertebrate groups may exhibit similar relationships. The pineal gland and the pineal gland secretory product (melatonin) are regarded as synchronizers of daily and seasonal rhythms to the external light-dark cycle in most vertebrates [5,6], including ectothermic vertebrates such as fish [7–9]. Regarding annual rhythmicity, seasonal peaks of lymphatic organ size and structure generally occur in late autumn or early winter, whereas smaller lymphatic organs are observed prior to the onset of breeding. Although many of the field data suggest that immune function and disease processes also become more prevalent during the winter, the opposite seasonal pattern has also been observed in some studies. Evidence for seasonal fluctuations in lymphatic organ size, structure, immune function, and disease processes and their possible interactions with recurrent environmental stressors has been reviewed by Nelson and Demas [10]. Furthermore, seasonality has also been shown to affect the immune response, immune competence, and the prevalence of disease in vertebrates [11,12]. Authors of previous studies have postulated that the seasonality associated with various immune parameters in fish may occur in response to seasonal increases in the level of potential pathogens in the aquatic environment. For example, several diseases of the aquatic environment present a seasonal pattern in carp, including proliferative kidney disease [13], furunculosis (*Aeromonas salmonicida*) [14], winter disease syndrome [15], and spring viraemia [16]. Fluctuations observed in immune activity could be indicative of the organism preparing for a potential attack by seasonal pathogens, and if this is the case, the change in climatic conditions must be anticipated; otherwise, any preventive measures to combat the increased pathogen load would likely occur too late. However, the mechanisms that regulate these seasonal variations have yet to be fully elucidated.

Many environmental challenges are recurrent and thus are predictable [11]. Animals could presumably enhance their survival and increase their fitness if they are able to immunologically anticipate challenging conditions such that they may cope with these seasonal health threats [17]. A potential mechanism to anticipate changes in the season may also be transmitted through the pineal hormone melatonin as an intermediary in the process [18].

Melatonin (*N*-acetyl-5-methoxytryptamine) is a hormone secreted by the pineal gland and the retina in vertebrates and is produced from the amino acid tryptophan [8]. Melatonin exhibits a strong daily circadian rhythm; the majority of the hormone is produced during the dark phase of the photoperiod. In fish, the effects of melatonin are mediated through high-affinity receptors [8,17,19–24]. Melatonin fluctuations in organisms suggest the involvement of melatonin in the circadian rhythm and implicate melatonin in the regulation of diverse behavioral and physiological events in vertebrates [21,25–27]. Notably, melatonin biosynthesis has also been described in human peripheral blood mononuclear leucocytes [28,29], mouse and human bone marrow cells [30], and human lymphocytes [31]. However, the physiological significance of immune cell-derived melatonin remains unclear, and studies analyzing immune cell-derived melatonin in fish have yet to be conducted.

In the 1990s, studies of the influence of seasonal changes on the fish immune system revealed that these changes promote health and facilitate survival [10,12]; however, the authors of these studies did not relate the results to melatonin or other rhythmicities of the organism. More recent studies have demonstrated that some fish immune parameters follow a circadian rhythm that is affected by the photoperiod and likely by melatonin levels [32]. Follow-up studies have since shown that melatonin affects the fish immune response *in vitro* and *in vivo*[33,34]. A description of the direct effects of melatonin on the main components of the fish immune system as well as indirect studies focusing on daily and seasonal rhythms, which imply the involvement of melatonin levels, is enumerated below. Notably, no studies regarding the effects of biological rhythms on the adaptive immune system are available, as all studies have investigated the effect of rhythmicity on innate/natural or nonspecific immune responses.

The present review summarizes the current knowledge suggesting that the relationship between melatonin and the fish immune system is an integral part of the fish endocrine-immune interaction profile. This review will focus mainly on teleosts of interest to ornamental or aquaculture practices.

## 2. Melatonin and Biological Rhythms in Fish

During an annual cycle, the aquatic environment is mainly affected by two primary factors, temperature and photoperiod, which are interlinked and follow very similar cycles. During the spring, water temperatures and light periods increase, while the opposite occurs in autumn. This seasonality affects the life history of fish with respect to the timing of developmental, behavioral, physiological, and maturation processes (such as locomotor activity, growth performance, smoltification, and reproduction), which are synchronized with seasonal changes in temperature, day length, and food supply [35]. During the winter months (when the daily dark photoperiod is long), melatonin is produced for a greater length of time relative to the shorter dark photoperiod of summer days in vertebrates [25]. These light-dark rhythms and the consequential changes in the level of melatonin in serum also influence many processes in fish such as sedation, skin pigmentation, oxygen consumption, osmoregulation, thermoregulation, food intake, shoaling behavior, and, as more recently described, immunity [7,8,32,36–41]. In addition to light, melatonin production in fish is also affected by water temperature. Higher levels of melatonin have been reported in fish maintained at a high temperature than in the same fish species maintained at lower temperatures [32]. Therefore, the immune response seems to be altered in daily/yearly cycles, and these changes appear to be related to water temperature.

In addition, a third parameter that has been shown to affect melatonin synthesis in fish is the lunar cycle. Although the seasonal and circadian rhythms have been fairly well characterized, little is known regarding the effects of the lunar cycle on the behavior and physiology of humans and animals. In fish, the lunar clock influences reproduction by affecting the regulation of the hypothalamus-pituitary-gonad axis. Previous studies have suggested that melatonin and endogenous steroids may mediate the described cyclic alterations in certain physiological processes [42]. Studies of the golden rabbit fish (*Siganus guttatus*), which spawns synchronously at approximately the first-quarter moon during the reproductive season, and the sea grass rabbit fish (*Siganus canaliculatus)* revealed daily fluctuations of melatonin levels in the blood, which were low during the day and high at night [43,44]. Furthermore, the plasma melatonin concentration during a new moon was higher than during a full moon, and when the fish were exposed to moonlight from a new moon or a full moon at midnight, the melatonin concentration decreased to control levels. These results suggest that the fish may perceive moonlight intensity as a cue to alter plasma melatonin levels according to the time of night [43,44].

Involvement of the light-dark cycle or melatonin levels in the regulation of the immune system has been extensively described in mammals [6,8,24] but has been rarely investigated in fish. Similarly, in general, endocrine-immune system interactions have been widely demonstrated in mammals. Further investigation of the role of photoperiod and temperature in melatonin levels will help to establish the roles of each of these factors in fish immunity.

## 3. A Brief Summary of Fish Immunity

During evolution, two general systems of immunity have emerged: innate/natural/nonspecific immunity and adaptive/acquired/specific immunity. The innate system is phylogenetically older and is found in some form in all multicellular organisms, whereas the adaptive system appeared approximately 450 million years ago and is found in all vertebrates except jawless fish [45]. The bony fishes are derived from one of the earliest divergent vertebrate lineages and possess both innate and acquired immune systems. Bony fishes are considered an ideal model for studying the underpinnings of the fish immune system because of their phylogenetic age and the fact that the adaptive immune system of bony fishes is not nearly as sophisticated as the mammalian adaptive immune system. Comparative studies of the innate immune system in invertebrates and early chordates can provide insight into the degree of homology between fish and mammals.

Teleosts, the modern branch of bony fishes, comprise the largest group of vertebrates, with more than 20,000 species. Teleosts also display considerable diversity and are thus found in all aquatic biota. The basal position of the teleosts in vertebrate phylogeny makes most teleosts attractive for genomic and functional comparative studies, particularly in studies of the immune system [46]. Fish possess an efficient and developed immunological response, and in the case of teleosts, the innate immune response represents the fundamental defense mechanism [47], whereas acquired responses are more limited when compared with the acquired responses found in higher vertebrates. Both types of responses have cellular and humoral compartments, and the relative importance of each compartment can change according to the age of the fish and multiple other factors. Although the rapid evolution of molecules involved in immunity often encumbers the identification of orthologous genes, the study of fish genomes has demonstrated that the essential components of the mammalian immune system are conserved in fish. The discovery of fundamental immune mechanisms present in fish reveals the primordial vertebrate immune repertoire, while the presence of unique adaptations illustrates how a population of organisms undergoing adaptive radiation may evolve by drawing on the available genomic resources in response to specific constraints [48].

Fish immune system organization presents specific characteristics because fish lack bone marrow and lymphatic nodules (see [49,50]). Thus, pronephros (anterior/head kidney) is the main lympho-haematopoietic tissue in fish and is the primary site for the development and production of B cells. The thymus is the main tissue responsible for the development and maturation of T cells, whereas the spleen is the main secondary lymphoid tissue in fish. Disperse leucocytes in the skin, gills, and gut form the mucosal associated-lymphoid tissue (MALT).

Regarding leucocyte types, fish lymphocytes are responsible for the production of antibodies (B cells) and the specific cellular immune response (T cells) in a manner that is reminiscent of the adaptive immune system of higher vertebrates. Monocyte-macrophages are leucocytes that display similar characteristics to both mammalian circulating monocytes and tissue macrophages. Granulocytes can be classified as neutrophils, eosinophils, and basophils according to their staining properties, but in the case of fish, the distribution and functions of the granulocytes do not mimic their mammalian counterparts as well as other components of the fish immune system. Monocyte-macrophages and some granulocytes form the phagocytic cells involved in the phagocytosis of particulate antigens and in the production of lytic enzymes and the respiratory burst reaction, in which very toxic reactive oxygen species (ROS) and nitrogen intermediates (RNI) are produced. Finally, nonspecific cytotoxic cells (NCCs) are involved in the lysis of tumor cells, virus-infected cells, and parasites in a fashion similar to mammalian natural killer (NK) cells. However, fish immune cells form a heterogeneous population (lymphocytes, granulocytes, and/or monocyte-macrophages), and therefore some authors refer to nonspecific cytotoxic activity in fish rather than cytotoxic activity specific to a cellular subtype or population.

The innate components of the humoral immune response are quite similar to those of other vertebrates (complement, lysozyme, interferons, interleukins, protease inhibitors, natural antibodies, pentraxins, and transferrin). The main differences in the humoral immune response are found in the adaptive components known as the immunoglobulins (Ig). Fish were previously thought to have only one immunoglobulin isoform, IgM, which is tetrameric instead of pentameric and harbors both membrane and soluble forms. However, recent studies have revealed the presence of other Ig isoforms (IgD, IgZ, and IgT) and have shed some light onto the complete repertoire of fish immunoglobulins and their evolution in vertebrates [51,52].

## 4. Effects of Rhythms and Melatonin on the Fish Immune System

### 4.1. Blood Cell Number

Some studies have identified variations in blood cell numbers according to circadian or annual rhythms. A slight seasonal effect was observed for red blood cell counts of rainbow trout, with the lowest and highest values recorded in the winter and summer, respectively (*Oncorhynchus mykiss*) [53]. The solubility of oxygen is higher in cold water than in warm water, suggesting that fewer red blood cells are needed to properly oxygenate fish in colder weather because oxygen is more readily available [54]. The higher solubility of oxygen in cold water could be a plausible explanation for the higher concentration of red blood cells in June and July [53]. Likewise, blood becomes more viscous at lower temperatures. To compensate for this increased viscosity, Antarctic fishes maintain fewer red blood cells [55], which may also help explain the lower concentration of red blood cells observed in rainbow trout during the winter months. Furthermore, winter is a period of reduced activity and thus less energy expenditure, i.e., reduced metabolism, reducing the amount of oxygen required by fish [53].

Likewise, leucocyte counts have also been shown to exhibit a seasonal rhythmicity in salmonids and in tench (*Tinca tinca*) [56–58]. Although the pattern of leucocyte counts reported in these studies differ, as a general trend, all leucocyte subtypes appear to be suppressed in the winter and increased in the summer, as was hypothesised by Slater and Schreck [56]. A reduction in circulating lymphocytes was also observed in gilthead seabream (*Sparus aurata*) grown at low water temperatures [59]. However, this study proposed that the changes in the lymphocyte population were due to variations in the water temperature, and the authors never related the changes to the light-dark cycle or melatonin production.

### 4.2. Innate Immunity

For many years, the degree of the innate immune response was assumed to remain constant throughout the year with no seasonal variations, such that the immune system provides a constant defense against invading pathogens [60]. However, several studies have since demonstrated a general seasonal influence on some immune parameters under different environmental conditions. When grown at low temperatures, fish displayed immunodepression, as manifested by a decrease in various immune parameters. Innate immunological indicators appear to be affected by low temperatures in rainbow trout (freshwater fish species) and gilthead seabream (seawater fish species) [53,61].

Many humoral factors and cellular activities have been investigated in studies of the fish innate immune system; however, very few studies have focused on the effects of melatonin on innate immune system factors or activities. The direct link between photoperiod and various fish immune parameters was described for the first time in both gilthead seabream and European sea bass (*Dicentrarchus labrax*) [32]. The specimens were exposed to a constant light-dark photoperiod (12 h light:12 h dark, from 08:00 to 20:00, with 08:00 as the zeitgeber time) and were sampled at different hours of the day. The results demonstrated a clear circadian rhythmicity of several immune parameters; this rhythmicity correlated with the daily pattern of melatonin levels because temperature was kept constant [32]. Since this study, our group has evaluated the direct effects of melatonin administration *in vitro* and *in vivo* on the fish innate immune response. These trials were performed based on available data showing that circulating melatonin levels range from 50 pM to 2 nM in gilthead seabream [40] and European sea bass [36,37]. As expected, in both species, the lowest levels of melatonin were detected at the beginning of the light phase, and the highest levels of melatonin were detected during the night. Isolated head kidney leucocytes of gilthead seabream and European sea bass were incubated with a wide range of melatonin concentrations spanning physiological and pharmacological levels (from 20 pM to 400 mM), and various innate immune parameters were analyzed [34]. In another study, seabream specimens were injected with 1 or 10 mg of melatonin/kg of biomass and were sampled after 1, 3, and 7 days to evaluate the circulating melatonin levels, humoral and cellular innate immune parameters, and the expression of several immune-relevant genes [33]. The injection of melatonin resulted in elevated circulating levels of melatonin beyond the levels observed during normal light-dark cycles.

#### 4.2.1. Complement

The complement system is composed of different serum proteins and represents a very important defense system in fish [61]. The complement system consists of a complex enzyme cascade comprising several inactive glycoproteins, which are activated by one of the three known activation routes: classical, alternative, and lectin-mediated [62,63]. The functions of the complement system include lytic activity (for viruses, bacteria, and parasites) and neutralization of bacterial exotoxins. The complement system also provides signals of potential pathogens to the host and contributes to the degradation of pathogens through the recruitment of immune cells and through opsonization [45].

The fish complement system is affected by biological rhythms and by exogenous melatonin. Preliminary studies reported that low temperatures correlate with a significant decrease in serum complement activity in gilthead seabream [59], but this correlation was observed only as a consequence of temperature changes and not as a response to light-dark cycles. More recently, we found that complement activity was higher during the light hours and lower during the dark hours in gilthead seabream and European sea bass fish species exposed to constant light-dark cycles [32]. However, the differences were only significant for the European sea bass. Further analysis of the seabream specimens injected with melatonin revealed no significant changes in complement activity [33]. Thus, results from studies in seabream demonstrate that low temperatures but not melatonin and photoperiod change complement activity initiated by the alternative pathway. Further studies of more fish species are needed to ascertain whether melatonin affects complement activity.

#### 4.2.2. Lysozyme

Approximately 90 years have passed since Alexander Fleming discovered the antimicrobial activity of lysozyme, the first natural antibiotic isolated from humans [64]. Low temperatures in gilthead seabream (resulting in low melatonin production) have been shown to correlate with a decrease in plasma lysozyme activity [59]. However, this correlation has not been observed in other species of fish. For example, when lysozyme activity was measured in Nile tilapia (*Oreochromis niloticus*) grown at four different temperatures (18.4 °C, 23 °C, 28 °C and 33 °C), the fish cultured at 33 °C exhibited a decrease in lysozyme activity after 4 weeks, which suggests that water temperature has a limited effect on lysozyme activity in this fish species [65]. A correlation between variations in the level of fish serum lysozyme and the season of the year has been demonstrated. In fact, lysozyme activity increases during the spring, peaks in late summer, and decreases in autumn, with its lowest level occurring in late winter. Thus, plasma lysozyme levels exhibit a seasonal pattern of activity during a 12-month period (very similar to the seasonal pattern for white blood cell counts) in the following fish species: rainbow trout, plaice (*Pleuronectes platessa*), sea trout (*Salmo trutta*), dab (*Limanda limanda*), Asian catfish (*Clarias batrachus*), and Atlantic halibut (*Hippoglossus hippoglossus*) [53,66–71].

Lysozyme activity also follows a clear daily rhythm in fish, although different inter-specific patterns were observed in seabream and sea bass [32]. In gilthead seabream, lysozyme activity increases to a maximum at 20:00 (start of dark or end of light), decreasing thereafter to basal levels at the end of the dark phase. However, in sea bass, serum lysozyme levels decreased during the light hours and increased during the dark hours, reaching the highest values at the end of the dark phase (08:00) [32]. No studies have evaluated lysozyme activity in other vertebrate groups or demonstrated a direct effect of melatonin on lysozyme activity.

#### 4.2.3. Peroxidase

Peroxidases [myeloperoxidase (MPO) and eosinophil peroxidase (EPO)] are very important microbicidal agents in mammals. Peroxidases are contained within the granules of phagocytic cells and are discarded into phagosomes or to the extracellular medium after phagocytosis, where peroxidases exert their lytic activity [72,73]. Peroxidase release into the blood has been used as an indicator of the immunologically active status of circulating leucocytes.

The limited available results on the influence of the photoperiod on serum peroxidase levels suggest that peroxidase secretion depends on the fish species. Peroxidase activity was significantly higher in seabream specimens sampled at 08:00 than during the rest of the daily cycle, whereas sea bass exhibited non-statistically significant variations in peroxidase activity relative to the photoperiod [32]. Moreover, after injection with melatonin, seabream exhibit persistently higher levels of serum peroxidase seven days after receiving a melatonin dose of 10 mg/kg [33]. These data suggest that in seabream, prolonged melatonin accumulation results in increased circulating peroxidase levels, which occur at the end of the dark phase, when peroxidase levels and activity are at a maximum.

Interestingly, the *in vitro* peroxidase activity of head kidney leucocytes derived from seabream and sea bass remained unchanged upon exposure to melatonin at concentrations ranging from 100 to 200 mM, and only a very high concentration (400 mM) resulted in a significant inhibition of peroxidase activity [34]. By contrast, melatonin injection resulted in increased peroxidase activity of head kidney leucocytes; this activity persisted for a short time and subsided after one day [33]. These differences between the *in vitro* and *in vivo* effects of melatonin exposure on the peroxidase activity of head kidney leucocytes may be due to the indirect action of melatonin on seabream leucocytes, as has been observed in other vertebrates [4,24]. Unfortunately, among vertebrates, the effect of melatonin on peroxidase activity has only been evaluated in fishes.

#### 4.2.4. Respiratory Burst

A respiratory burst (also called an oxidative burst) is the rapid release of reactive oxygen species (superoxide radicals and hydrogen peroxide) from different cell types. The respiratory burst plays an important role in the immune system and is critical for the phagocytic degradation of internalized particles.

Human neutrophils increase their respiratory burst at a melatonin concentration of 10 nM, but the respiratory burst is inhibited at 2 mM, suggesting a dose-dependent biphasic mechanism [74]. Moreover, in birds, although physiological and supraphysiological melatonin doses increase avian phagocytic activity, the respiratory burst is consistently decreased [75,76].

Early studies in rainbow trout demonstrated that the head kidney leucocyte respiratory burst seems to be temperature dependent as an increase in temperature increased respiratory burst activity [77]. However, temperature had no effect on the respiratory burst activity of blood leucocytes in Atlantic cod (*Gadus morhua*) [65]. Although significant differences in the monthly respiratory burst values of rainbow trout head kidney macrophages were observed, these differences could not be proven as seasonal changes because the highest levels of respiratory burst activity occurred in August and February and the lowest level occurred in January [53]. The lack of an influence of the season on respiratory burst was previously documented in a study on croaker (*Micropogonias furnieri*), which also suggested that the respiratory burst activity was not affected by season [78].

*In vitro* studies analyzing the effects of melatonin in fish suggested that the incubation of sea bass leucocytes with melatonin at a concentration of 400 nM or higher results in increased respiratory burst activity in a dose-dependent manner but does not affect the respiratory burst activity of seabream leucocytes [34]. Interestingly, the intraperitoneal injection of melatonin results in increased respiratory burst activity of seabream head kidney leucocytes after one day, but respiratory burst activity returned to control levels thereafter [33]. These data confirm that there are inherent differences in the effects of melatonin *in vitro* and *in vivo* as well as between different fish species.

#### 4.2.5. Phagocytosis

Phagocytosis, the process by which cells engulf, kill, and digest different particles (damaged or altered cells, microorganisms, *etc*.), plays an important role in the non-specific immune response [79]. *In vitro*, physiological (0.1 and 1 nM) or pharmacological (100 mM) melatonin concentrations have been shown to increase phagocytosis in birds [80] and mammals [80]. Moreover, the phagocytes in mammals and birds have been reported to exhibit day-night variations in their phagocytic activity [81–84], as phagocytosis increases during night hours and coincides with elevated melatonin levels.

One study reported evidence of diurnal rhythmicity in splenic phagocytic activity in a freshwater fish species (*Channa punctatus*) characterized by increased phagocytic activity during the light phase relative to the dark phase; the rhythmicity of the phagocytic activity was inhibited by exogenous melatonin administration [85]. Furthermore, the direct involvement of melatonin in the modulation of phagocytosis was demonstrated in *in vitro* experiments in which an irreversible tryptophan hydroxylase inhibitor (para-chlorophenylalanine, pCPA) known to diminish melatonin biosynthesis [86] was administered to fish. pCPA reversed the typical decrease in phagocytic activity observed during the dark phase, and phagocytic activity could be restored upon melatonin administration [85]. Thus, melatonin appears to suppress phagocytic activity in a dose-dependent manner without affecting the viability of *C. punctatus* phagocytes [85], in contrast to reports in endothermic vertebrates that have shown that melatonin possesses immunoenhancing effects [4,80,87]. By contrast, *in vitro* incubation of seabream and sea bass head kidney leucocytes with melatonin had no effect on phagocytic activity [34], whereas a small but significant increase in the *in vivo* phagocytic activity of seabream leucocytes was observed after melatonin injection [33].

Furthermore, the existence of functional membrane-bound melatonin receptors on fish phagocytes was pharmacologically demonstrated by adding luzindole (a melatonin membrane receptor antagonist), which completely blocked the inhibitory effect of melatonin on phagocytic activity of isolated splenic macrophages [85]. This study also demonstrated that the receptor-coupled adenylate cyclase-protein kinase A (PKA) pathway is involved in the transduction of melatonin signaling, as both adenylate cyclase and PKA inhibitors completely nullified melatonin-induced suppression of phagocytic activity. Increased intracellular cAMP levels in response to melatonin further demonstrated that cAMP serves as the secondary messenger for downstream melatonin signaling. However, manipulation of phospholipase C/PKC failed to influence the effects of melatonin on phagocytic activity. Collectively, these observations in *C. punctatus* suggest that melatonin regulates the diurnal rhythmicity of phagocytic activity in fish and mediates these effects through membrane-bound receptors coupled to the cAMP-PKA pathway [85].

#### 4.2.6. Cell-Mediated Cytotoxicity

Cell-mediated cytotoxic activity is conducted by natural killer (NK) cells or by cytotoxic T lymphocytes in mammals, whereas a heterogeneous population of lymphocytes, monocyte-macrophages, and/or granulocytes performs an analogous task in fish. Cell-mediated cytotoxic activity is increased upon melatonin administration *in vivo* but is inhibited after *in vitro* incubation of cells with melatonin [88].

In gilthead seabream, *in vitro* administration of melatonin did not affect the innate cytotoxic activity of head kidney leucocytes, whereas intraperitoneal injection produced a significant increase in innate cytotoxic activity 1 and 3 days post-injection [33,34].

### 4.3. Adaptive Immunity

The photoperiod has been shown to affect the mammalian adaptive immune system, antigen-specific primary and secondary humoral immunity [89], and the efficiency of immune system sensitization [90]. Adaptive immunity in fish has also been shown to exhibit a seasonal cycle over a 12-month period with specific changes in the resting antibody titer, the ability to respond to antigenic challenge [91], and the lymphoid system [92,93]. Unfortunately, no other studies have been conducted on this topic.

### 4.4. Expression of Immune-Relevant Genes

In mammals, in addition to increased leucocyte activity, melatonin induces higher levels of interleukin (IL)-1β, IL-2, IL-3, IL-6, interferon-gamma (IFNγ), major histocompatibility complex (MHC) class II, tumor necrosis factor-α, macrophage colony-stimulating factor, transforming growth factor-β, stem cell factor, tissue factor, and the melatonin-induced opioid system at either the transcript or the protein level [4]. In agreement with these reports, seabream injected with melatonin have been shown to up-regulate head kidney gene expression of IL-1β, MHC, and interferon-regulatory factor (IRF)-1 at 1 and 3 days post-injection, but these genes were slightly down-regulated thereafter, suggesting the existence of a biphasic response [33]. Interestingly, the most up-regulated gene in seabream was IRF-1, most likely due to the beneficial effect of melatonin on seabream cytotoxic activity, similar to the melatonin-mediated release of IFNγ by mammalian NK cells. By contrast, no differences in T- and B-lymphocyte markers (TCR and Ig, respectively) were observed at the transcript level, indicating a lack of an effect of melatonin on lymphocyte activation or proliferation [33]. However, further studies in fish are needed to clarify the role of these genes in immunity and how they are regulated by melatonin.

## 5. Mechanisms by Which Melatonin Regulates Fish Immune Function

The mechanism(s) by which melatonin regulates immune function remain unresolved. Three possible mechanisms of action have been proposed in mammals: a direct action mediated by specific cell membrane and nuclear receptors present in different immune cell types [94,95], an indirect effect through the release of lymphokines, steroid hormones, glucocorticoids, prolactin, and opioids [4,96], or a combination of both models. Several hypotheses have been proposed to explain the contradictory results published thus far (see [4]) regarding the *in vitro* and *in vivo* effects of melatonin on immune cell activity: (a) the effect of melatonin on immune cells is mediated via other tissues, cells, hormones, and/or cytokines that are not present in culture media; (b) leucocytes may contain low numbers of specific melatonin receptors and/or these receptors could be very low affinity; (c) leucocytes may produce and release melatonin into the culture medium, which could mask the effects of exogenous melatonin. Melatonin receptors may be of low (MT3, a putative melatonin membrane receptor, also known as ML2, which belongs to the quinone reductase family, although its physiological role remains unknown [97]) or high (MT1, MT2 and Mel1c) affinity. MT3 is found in mammalian species, MT1 and MT2 are found in all vertebrates, whereas Mel1c is found in non-mammalian species. The melatonin receptors have been cloned, and their functional properties have been assayed in several fish species. Melatonin receptors are found in tissues such as the brain, pineal gland, pituitary gland, retina, gills, kidneys, liver, and skin [97]. Thus, partial or full-length sequences for the three receptor subtypes, Mel1a (MT1), Mel1b (MT2), and Mel1c, have been obtained for zebrafish (*Danio rerio*) [98], Senegalese sole (*Solea senegalensis*) [99], goldfish (*Carassius auratus*) [100], and European sea bass [101]. Partial or full-length sequences have been obtained for Mel1a and Mel1b for rainbow trout [102] and pike (*Esox lucius*) [103], and partial or full-length sequences have been obtained for Mel1a and Mel1c for rabbit fish [104,105]. Interestingly, the melatonin receptor Mel1b has recently been discovered on the surface of blood cells of European sea bass [101], suggesting the possible involvement of melatonin in the regulation of blood cell immune responses. This observation is supported by the demonstration of melatonin receptors on the surface of splenic macrophages of *C. punctatus*, although the specific melatonin receptor subtype has not been confirmed [85]. Further studies are needed to clearly demonstrate the presence and abundance of melatonin receptors on fish leucocytes and their involvement in the immune response. The results from these studies could confirm whether the effects of melatonin on fish leucocytes are direct and/or indirect.

The density of melatonin receptor binding sites follows a circadian rhythm in goldfish in which they peak during the dark phase but maintain a constant affinity (Kd) [106]. In masu salmon (*Oncorhynchus masou*) and pike, receptor density and affinity follow a circadian rhythm [107,108], whereas in European sea bass, melatonin receptor density and affinity peak at opposite phases [97], as the highest melatonin receptor activity correlated with the lowest hormone concentration. In mammals and birds, the Kd of the melatonin receptors allows the appropriate cells to respond to physiological concentrations of melatonin at night, suggesting that melatonin receptors require higher levels of melatonin to be active. The lowest melatonin receptor Kd was observed in lymphocytes, followed by macrophages, and the highest Kd was observed in granulocytes (in the mM range) (see [4]). Seabream cytotoxic activity may be the immune parameter most enhanced by melatonin because it is mediated by lymphocytes and phagocytes (acidophils and macrophages), whereas the remaining cellular innate immune activities analyzed are mediated predominantly by phagocytes [33], which require much higher levels of melatonin for activation.

Furthermore, the expression of genes encoding different hormones (e.g., growth hormone, prolactin, somatolactin, and cortisol) and their cognate receptors also exhibit seasonal changes [109–111] in fish, which is likely because these hormones display changes during daily cycles and could influence daily rhythms of the immune system. In this sense, a direct link between melatonin and glucocorticoid biology has been established in mice, as melatonin enhances immune function (melatonin enhances the antibody response via an opiatergic mechanism), whereas glucocorticoid levels suppress immune function [112].

The influence of other hormones on the effects of melatonin on the fish immune system could also be affected by many other environmental conditions, such as the presence of copper (Cu). Chronic sub-lethal exposure to Cu causes important cellular and physiological changes in fish that enable fish to survive. Cu affects specific neuroendocrine functions, including the loss of circadian rhythm. Dietary Cu exposure causes a failure to respond to circulating melatonin and a decrease in circulating serotonin [113]. Rainbow trout exposed to 730 mg Cu/kg food for 3 months exhibit Cu-dependent loss of circadian rhythm [114]. Periods of high locomotor activity are normally associated with increased melatonin secretion from the pineal gland and elevated plasma cortisol [115,116]. In several fish species, copper does not prevent the secretion of melatonin as normal but causes a loss of locomotor activity [114], suggesting that the fish are not able to respond to melatonin. Collectively, these data could be explained in the context of some *in vitro* data that suggest that melatonin is chelated by Cu [117].

## 6. Concluding Remarks and Future Research

One of the main conclusions of this review is that much work remains to obtain a complete understanding of the relationship between melatonin and the fish immune system. At present, the effects of melatonin on some activities of the fish immune system remain poorly understood. The limited results available are insufficient to identify a common pattern of responses of the immune system to exposure to high or low levels of melatonin. More studies are needed to confirm pineal gland-immune system communication and to demonstrate whether circadian rhythm-mediated immune responses correlate with an increase in disease resistance.

Taken together, the pattern of circadian rhythmicity of immune responses seems to be dependent on the species studied, the strain of animal, and the type of immune factors or cells and their specific functions. Knowledge of daily fluctuations in immune function may help to improve our understanding of the possibilities and constraints of fish melatonin immunotherapy. Furthermore, studies regarding melatonin seasonal rhythms on fish immune activity are required. These data could be of great interest to fish farmers, who could use this knowledge to improve the immunity of fish in aquaculture and minimise the adverse effects caused by the time of year through the use of products such as immunostimulants and probiotics, which have become fashionable in recent years.

The effects of melatonin on numerous humoral immune components, such as lectins, antimicrobial peptides (AMPs), and interferons, have not been addressed. For example, over the past two decades, more than 1200 AMPs have been identified or predicted from various organisms [64]. Moreover, understanding whether melatonin biosynthesis occurs in fish blood leucocytes and in head kidney haemopoietic cells, as described in human peripheral blood mononuclear leucocytes and bone marrow cells, is important as this phenomenon could be of great physiological significance.

In summary, many more studies are needed to clarify the role of melatonin in immune function as well as potential relationships between melatonin and other hormones or life cycle parameters. In addition, increasing fish age may affect melatonin levels similarly to age in mammals, which generally exhibit declines in pineal gland function and plasma melatonin concentration with age [118]. Studies regarding the effects of age on circadian function in fish could be relevant to broodstock management.

A better knowledge of fish melatonin and immune system interactions will facilitate the development of novel practical indications (such as photoperiod manipulation) for enhancing the immune system and welfare of fish in hatcheries and growing farms. Future research will provide greater insight into the role of melatonin.

## References

[1] Food and Agriculture Organization of the United Nations (FAO) (2012). The State of World Fisheries and Aquaculture.

[2] Balcázar J.L., de Blas I., Ruiz-Zarzuela I., Cunningham D., Vendrell D., Muzquiz J.L. (2006). The role of probiotics in aquaculture. Vet. Microbiol.

[3] Zhu L.Y., Nie L., Zhu G., Xiang L.X., Shao J.Z. (2013). Advances in research of fish immune-relevant genes: A comparative overview of innate and adaptive immunity in teleosts. Dev. Comp. Immunol.

[4] Guerrero J.M., Reiter R.J. (2002). Melatonin-immune system relationships. Curr. Top. Med. Chem.

[5] Cassone V.M. (1998). Melatonin’s role in vertebrate circadian rhythms. Chronobiol. Int.

[6] Falcón J. (1999). Cellular circadian clocks in the pineal. Prog. Neurobiol.

[7] Ekström P., Meissl H. (1997). The pineal organ of teleost fishes. Rev. Fish Biol. Fish.

[8] Falcón J. (2007). Nocturnal melatonin synthesis: How to stop it. Endocrinology.

[9] Okimoto D.K., Stetson M.H. (1999). Presence of an intrapineal circadian oscillator in the teleostean family *Poeciliidae*. Gen. Comp. Endocrinol.

[10] Nelson R.J., Demas G.E. (1996). Seasonal changes in immune function. Quart. Rev. Biol.

[11] Bowden T.J., Thompson K.D., Morgan A.L., Gratacap R.M., Nikoskelainen S. (2007). Seasonal variation and the immune response: A fish perspective. Fish Shellfish Immunol.

[12] Zapata A.G., Varas A., Torroba M. (1992). Seasonal variations in the immune system of lower vertebrates. Immunol. Today.

[13] Hedrick R.P., MacConnell E., de Kinkelin P. (1993). Proliferative kidney disease of salmonid fish. Ann. Rev. Fish Dis.

[14] Hjeltnes B., Bergh O., Wergeland H., Holm J.C. (1995). Susceptibility of Atlantic cod *Gadus morhua*, halibut *Hippoglossus hippoglossus* and wrasse (*Labridae*) to *Aeromonas salmonicida* subsp. *Salmonicida* and the possibility of transmission of *Furunculosis* from farmed salmon *Salmo salar* to marine fish. Dis. Aquat. Org.

[15] Gallardo M.A., Sala-Rabanal M., Ibarz A., Padrós F., Blasco J., Fernández-Borrás J., Sánchez J. (2003). Functional alterations associated with “winter syndrome” in gilthead sea bream (*Sparus aurata*). Aquaculture.

[16] Padhi A., Verghese B. (2012). Molecular evolutionary and epidemiological dynamics of a highly pathogenic fish rhabdovirus, the spring viremia of carp virus (SVCV). Vet. Microbiol.

[17] Nelson R.J., Demas G.E., Klein S.L., Kriegsfeld L.J. (1995). The influence of season, photoperiod, and pineal melatonin on immune function. J. Pineal Res.

[18] Taylor J.F., North B.P., Porter M.J.R., Bromage N.R., Migaud H. (2006). Photoperiod can be used to enhance growth and improve feeding efficiency in farmed rainbow trout, *Oncorhynchus mykiss*. Aquaculture.

[19] Reiter R.J., Miles A., Philbrick D.R.S., Thompson C. (1988). Neuroendocrinology of Melatonin. Melatonin: Clinical Perspectives.

[20] Porter M (1996). The Role of Melatonin and the Pineal Gland in the Photoperiodic Control of Reproduction and Smoltification in Salmonid Fish. Ph.D. Thesis.

[21] Liebmann P.M., Wolfler A., Felsner P., Hofer D., Schauenstein K. (1997). Melatonin and the immune system. Int. Arch. Allergy Immunol.

[22] Nelson R.J., Drazen D.L. (1999). Melatonin mediates seasonal adjustments in immune function. Reprod. Nutr. Dev.

[23] Hazlerigg D.G. (2001). What is the role of melatonin within the anterior pituitary?. J. Endocrinol.

[24] Carrillo-Vico A., Guerrero J.M., Lardone P.J., Reiter R.J. (2005). A review of the multiple actions of melatonin on the immune system. Endocrine.

[25] Randall C.F., Bromage N.R., Thorpe J.E., Miles M.S., Muir J.S. (1995). Melatonin rhythms in Atlantic salmon (*Salmo salar*) maintained under natural and out-of-phase photoperiods. Gen. Comp. Endocrinol.

[26] Moore C.B., Siopes T.D. (2003). Melatonin enhances cellular and humoral immune responses in the Japanese quail (*Coturnix coturnix japonica*) via an opiatergic mechanism. Gen. Comp. Endocrinol.

[27] Iigo M., Fujimoto Y., Gunji-Suzuki M., Yokosuka M., Hara M., Ohtani-Kaneko R., Tabata M., Aida K., Hirata K. (2004). Circadian rhythm of melatonin release from the photoreceptive pineal organ of a teleost, ayu (*Plecoglossus altivelis*) in flow-thorough culture. J. Neuroendocrinol.

[28] Finocchiaro L.M., Arzt E.S., Fernandez-Castelo S., Criscuolo M., Finkielman S., Nahmod V.E. (1988). Serotonin and melatonin synthesis in peripheral blood mononuclear cells: Stimulation by interferon-gamma as part of an immunomodulatory pathway. J. Interferon Res.

[29] Finocchiaro L.M., Nahmod V.E., Launay J.M. (1991). Melatonin biosynthesis and metabolism in peripheral blood mononuclear leucocytes. Biochem. J.

[30] Conti A., Conconi S., Hertens E., Skwarlo-Sonta K., Markowska M., Maestroni J.M. (2000). Evidence for melatonin synthesis in mouse and human bone marrow cells. J. Pineal Res.

[31] Carrillo-Vico A., Calvo J.R., Abreu P., Lardone P.J., Garcia-Maurino S., Reiter R.J., Guerrero J.M. (2004). Evidence of melatonin synthesis by human lymphocytes and its physiological significance: Possible role as intracrine, autocrine, and/or paracrine substance. FASEB J.

[32] Esteban M.A., Cuesta A., Rodríguez A., Meseguer J. (2006). Effect of photoperiod on the fish innate immune system: A link between fish pineal gland and the immune system. J. Pineal Res.

[33] Cuesta A., Cerezuela R., Esteban M.A., Meseguer J. (2008). *In vivo* actions of melatonin on the innate immune parameters in the teleost fish gilthead seabream. J. Pineal Res.

[34] Cuesta A., Rodríguez A., Calderón M.V., Meseguer J., Esteban M.A. (2007). Effect of the pineal hormone melatonin on teleost fish phagocyte innate immune responses after *in vitro* treatment. J. Exp. Zool. A Ecol. Genet. Physiol.

[35] Bromage N.R., Porter M.J.R., Randall C. (2001). The environmental regulation of maturation in farmed finfish with special reference to the role of photoperiod and melatonin. Aquaculture.

[36] Bayarri M.J., Madrid J.A., Sánchez-Vázquez F.J. (2002). Influence of light intensity, spectrum and orientation on sea bass plasma and ocular melatonin. J. Pineal Res.

[37] Bayarri M.J., Rodríguez L., Zanuy S., Madrid J.A., Sánchez-Vázquez F.J., Kagawa H., Okuzawa K., Carrillo M. (2004). Effect of photoperiod manipulation on the daily rhythms of melatonin and reproductive hormones in caged European sea bass (*Dicentrarchus labrax*). Gen. Comp. Endocrinol.

[38] Vera L.M., López-Olmeda J.F., Bayarri M.J., Madrid J.A., Sánchez-Vázquez F.J. (2005). Influence of light intensity on plasma melatonin and locomotor activity rhythms in tench. Chronobiol. Int.

[39] Kleszczynska A., Vargas-Chacoff L., Gozdowska M., Kalamarz H., Martínez-Rodríguez G., Mancera J.M., Kulczykowska E. (2006). Arginine vasotocin, isotocin and melatonin responses following acclimation of gilthead sea bream (*Sparus aurata*) to different environmental salinities. Comp. Biochem. Physiol. A Mol. Integr. Physiol.

[40] Kulczykowska E., Kalamarz H., Warne J.M., Balment R.J. (2006). Day-night specific binding of 2-[125I]iodomelatonin and melatonin content in gill, small intestine and kidney of three fish species. J. Comp. Physiol. B.

[41] López-Olmeda J.F., Bayarri M.J., Rol de Lama M.A., Madrid J.A., Sánchez-Vázquez F.J. (2006). Effects of melatonin administration on oxidative stress and daily locomotor activity patterns in goldfish. J. Physiol. Biochem.

[42] Zimecki M. (2006). The lunar cycle: Effects on human and animal behavior and physiology. Postepy Hig. Med. Dosw.

[43] Rahman M.S., Morita M., Takemura A., Takano K. (2003). Hormonal changes in relation to lunar periodicity in the testis of the forktail rabbitfish, *Siganus argenteus*. Gen. Comp. Endocrinol.

[44] Takemura A., Susilo E.S., Rahman M.D., Morita M. (2004). Perception and possible utilization of moonlight intensity for reproductive activities in a lunar-synchronized spawner, the golden rabbitfish. J. Exp. Zool. A Comp. Exp. Biol.

[45] Zarkadis I.K., Mastellos D., Lambris J.D. (2001). Phylogenetic aspects of the complement system. Dev. Comp. Immunol.

[46] Magor B.G., Magor K.E. (2001). Evolution of effectors and receptors of innate immunity. Dev. Comp. Immunol.

[47] Magnadottir B. (2006). Innate immunity of fish (overview). Fish Shellfish Immunol.

[48] Levraud J.P., Boudinot P. (2009). The immune system of teleost fish. Med. Sci (Paris).

[49] Cuesta A., Meseguer J., Esteban M.A., Stoytcheva M. (2011). Immunotoxicological Effects of Environmental Contaminants in Teleost Fish Reared for Aquaculture. Pesticides in the Modern World - Risks and Benefits.

[50] Manning M.J., Black K.D., Pickering A.D. (1998). Immune Defence Systems. Biology of Farmed Fish.

[51] Hikima J., Jung T.S., Aoki T. (2010). Immunoglobulin genes and their transcriptional control in teleosts. Dev. Comp. Immunol.

[52] Hsu E., Criscitiello M.F. (2006). Diverse immunoglobulin light chain organizations in fish retain potential to revise B cell receptor specificities. J. Immunol.

[53] Morgan A.L., Thompson K.D., Auchinachie N.A., Migaud H. (2008). The effect of seasonality on normal haematological and innate immune parameters of rainbow trout *Oncorhynchus mykiss* L. Fish Shellfish Immunol.

[54] Stolen J.S., Draxler S., Nagle J.J. (1984). A comparison of temperature mediated immunomodulation between two species of flounder. Immunol. Comm.

[55] Graham M.S., Fletcher G.L., Haedrich R.L. (1985). Blood viscosity in arctic fishes. J. Exp. Zool.

[56] Slater C.H., Schreck C.B. (1998). Season and physiological parameters modulate salmonid leucocyte androgen receptor affinity and abundance. Fish Shellfish Immunol.

[57] Collazos M.E., Ortega E., Barriga C., Rodríguez A.B. (1998). Seasonal variation in haematological parameters in male and female *Tinca tinca*. MolCellBiochem.

[58] De Pedro N., Guijarro A.I., López-Patiño M.A., Martínez-Álvarez R., Delgado M.J. (2005). Daily and seasonal variations in haematological and blood biochemical parameters in the tench, *Tinca tinca* Linnaeus, 1758. Aquac. Res.

[59] Tort L., Rotllant J., Rovira L. (1998). Immunological suppression in gilthead sea bream *Sparus aurata* of the North West Mediterranean at low temperature. Comp. Biochem. Physiol. A Mol. Integr. Physiol.

[60] Ellis A.E. (2001). Innate host defense mechanisms of fish against viruses and bacteria. Dev. Comp. Immunol.

[61] Sakai D.K. (1992). Repertoire of complement in immunological defense mechanisms of fish. Annu. Rev. Fish Dis.

[62] Holland M.C., Lambris J.D. (2002). The complement system in teleosts. Fish Shellfish Immunol.

[63] Nonaka M., Smith S.L. (2000). Complement system of bony and cartilaginous fish. Fish Shellfish Immunol.

[64] Nakatsuji T., Gallo R.L. (2012). Antimicrobial peptides: Old molecules with new ideas. J. Invest. Dermatol.

[65] Domínguez M., Takemura A., Tsuchiya M. (2005). Effects of changes in environmental factors on the non-specific immune response of Nile tilapia, *Oreochromis niloticus* L. Aquac. Res.

[66] Fletcher T.C., White A. (1976). The lysozyme of the plaice *Pleuronectes platessa* L. Comp. Biochem. Physiol. B.

[67] Muona M., Soivio A. (1992). Changes in plasma lysozyme and blood leucocyte levels of hatchery-reared Atlantic salmon (*Salmo salar* L.) and sea trout (*Salmo trutta* L.) during parr esmolt transformation. Aquaculture.

[68] Hutchinson T.H., Manning M.J. (1996). Seasonal trends in serum lysozyme activity and total protein concentrations in dab (*Limanda limanda* L.) samples from Lyme Bay, U.K. Fish Shellfish Immunol.

[69] Langston A.L., Hoare R., Stefansson M., Fitzgerald R., Wergeland H., Mulcahy M. (2002). The effect of temperature on non-specific defence parameters of three strains of juvenile Atlantic halibut (*Hippoglossus hippoglossus* L.). Fish Shellfish Immunol.

[70] Bowden T.J., Butler R., Bricknell I.R. (2004). Seasonal variation of serum lysozyme levels in Atlantic halibut (*Hippoglossus hippoglossus* L.). Fish Shellfish Immunol.

[71] Kumari J., Sahoo P.K., Swain T., Sahoo A.K., Sahu B.R., Mohanty B.R. (2006). Seasonal variation in the innate immune parameters of the Asian catfish *Clarias batrachus*. Aquaculture.

[72] Torreilles J., Guérin M.C., Roch P. (1996). Espèces oxygénés reactives et systèmes de defense des bivalves marins. CR Acad. Sci.

[73] Rodríguez A., Esteban M.A., Meseguer J. (2003). Phagocytosis and peroxidase release by seabream (*Sparus aurata* L.) leucocytes in response to yeast cells. Anat. Rec. A Discov. Mol. Cell. Evol. Biol.

[74] Pieri C., Recchioni R., Moroni F., Marcheselli F., Marra M., Marinoni S., di Primio R. (1998). Melatonin regulates the respiratory burst of human neutrophils and their depolarization. J. Pineal Res.

[75] Rodríguez A.B., Ortega E., Lea R.W., Barriga C. (1997). Melatonin and the phagocytic process of heterophils from the ring dove (*Streptopelia risoria*). Mol. Cell. Biochem.

[76] Rodríguez A.B., Terrón M.P., Duran J., Ortega E., Barriga C. (2001). Physiological concentrations of melatonin and corticosterone affect phagocytosis and oxidative metabolism of ring dove heterophils. J. Pineal Res.

[77] Nikoskelainen S., Bylund G., Lilius E.M. (2004). Effect of environmental temperature on rainbow trout (*Oncorhynchus mykiss*) innate immunity. Dev. Comp. Immunol.

[78] Amado L.L., da Rosa C.E., Leite A.M., Moraes L., Pires W.V., Pinho G.L., Martins C.M., Robaldo R.B., Nery L.E., Monserrat J.M. (2006). Biomarkers in croakers *Micropogonias furnieri* (Teleostei: *Sciaenidae*) from polluted and non-polluted areas from the Patos Lagoon estuary (Southern Brazil): Evidences of genotoxic and immunological effects. Mar. Pollut. Bull.

[79] Terrón M.P., Cubero J., Barriga C., Ortega E., Rodríguez A.B. (2003). Phagocytosis of *Candida albicans* and superoxide anion levels in ring dove (*Streptopelia risoria*) heterophils: Effect of melatonin. J. Neuroendocrinol.

[80] Pawlak J., Singh J., Lea R.W., Skwarlo-Sonta K. (2005). Effect of melatonin on phagocytic activity and intracellular free calcium concentration in testicular macrophages from normal and streptozotocin-induced diabetic rats. Mol. Cell. Biochem.

[81] Rodríguez A.B., Marchena J.M., Nogales G., Duran J., Barriga C. (1999). Correlation between the circadian rhythm of melatonin, phagocytosis, and superoxide anion levels in ring dove heterophils. J. Pineal Res.

[82] Barriga C., Martin M.I., Tabla R., Ortega E., Rodríguez A.B. (2001). Circadian rhythm of melatonin, corticosterone and phagocytosis: Effect of stress. J. Pineal Res.

[83] Berger J., Slapnicková M. (2003). Circadian structure of rat neutrophil phagocytosis. Comp. Clin. Pathol.

[84] Hriscu M. (2004). Circadian phagocytic activity of neutrophils and its modulation by light. J. Appl. Biomed.

[85] Roy B., Singh R., Kumar S., Rai U. (2008). Diurnal variation in phagocytic activity of splenic phagocytes in freshwater teleost *Channa punctatus*: Melatonin and its signaling mechanism. J. Endocrinol.

[86] Carrillo-Vico A., Lardone P.J., Fernández-Santos J.M., Martin-Lacave I., Calvo J.R., Karasek M., Guerrero J.M. (2005). Human lymphocyte-synthesized melatonin is involved in the regulation of the interleukin-2/interleukin-2 receptor system. J. Clin. Endocrinol. Metab.

[87] Terrón M.P., Cubero J., Marchena J.M., Barriga C., Rodríguez A.B. (2002). Melatonin and aging: *In vitro* effect of young and mature ring dove physiological concentrations of melatonin on the phagocytic function of heterophils from old ring dove. Exp. Gerontol.

[88] Lissoni P., Marelli O., Mauri R., Resentini M., Franco P., Esposti D., Esposti G., Fraschini F., Halberg F., Sothern R.B. (1986). Ultradian chronomodulation by melatonin of a Placebo effect upon human killer cell activity. Chronobiologia.

[89] Bilbo S.D., Dhabhar F.S., Viswanathan K., Saul A., Nelson R.J. (2003). Photoperiod affects the expression of sex and species differences in leukocyte number and leukocyte trafficking in congeneric hamsters. Psychoneuroendocrinology.

[90] Prendergast B.J., Bilbo S.D., Dhabhar F.S., Nelson R.J. (2004). Effects of photoperiod history on immune responses to intermediate day lengths in Siberian hamster (*Phodopus sungorus*). J. Neuroimmunol.

[91] Nakanishi T. (1986). Seasonal changes in the humoral immune response and the lymphoid tissues of the marine teleost, *Sebastiscus marmoratus*. Vet. Immunol. Immunopathol.

[92] Álvarez F., Razquín B.E., Villena A.J., Zapata A.G. (1998). Seasonal changes in the lymphoid organs of wild brown trout, *Salmo trutta* L: A morphometrical study. Vet. Immunol. Immunopathol.

[93] Wojtowicz A., Plytycz B. (1997). Seasonal changes of the gutassociated lymphoid tissue in the common toad, *Bufo bufo*. J. Nutr. Immunol.

[94] Carrillo-Vico A., García-Perganeda A., Naji L., Calvo J.R., Romero M.P., Guerrero J.M. (2003). Expression of membrane and nuclear melatonin receptor mRNA and protein in the mouse immune system. Cell. Mol. Life Sci.

[95] Pozo D., García-Maurino S., Guerrero J.M., Calvo J.R. (2004). mRNA expression of nuclear receptor RZR/RORalpha, melatonin membrane receptor MT, and hydroxindole-O-methyltransferase in different populations of human immune cells. J. Pineal Res.

[96] Bartness T.J., Powers J.B., Hastings M.H., Bittman E.L., Goldman B.D. (1993). The timed infusion paradigm for melatonin delivery: What has it taught us about the melatonin signal, its reception, and the photoperiodic control of seasonal responses?. J. Pineal Res.

[97] Bayarri M.J., Iigo M., Muñoz-Cueto J.A., Isorna E., Delgado M.J., Madrid J.A., Sánchez-Vázquez F.J., Alonso-Gómez A.L. (2004). Binding characteristics and daily rhythms of melatonin receptors are distinct in the retina and the brain areas of the European sea bass retina (*Dicentrarchus labrax*). Brain Res.

[98] Reppert S.M., Weaver D.R., Cassone V.M., Godson C., Kolakowski L.F. (1995). Melatonin receptors are for the birds: Molecular analysis of two receptor subtypes differentially expressed in chick brain. Neuron.

[99] Confente F., Rendon M., Besseau L., Falcon J., Muñoz-Cueto J.A. (2010). Melatonin receptors in a pleuronectiform species, *Solea senegalensis*: Cloning, tissue expression, day-night and seasonal variations. Gen. Comp. Endocrinol.

[100] Ikegami T., Azuma K., Nakamura M., Suzuki N., Hattori A., Ando H. (2009). Diurnal expressions of four subtypes of melatonin receptor genes in the optic tectum and retina of goldfish. Comp. Biochem. Physiol. A Mol. Integr. Physiol.

[101] Sauzet S., Besseau L., Herrera Perez P., Coves D., Chatain B., Peyric E., Boeuf G., Muñoz-Cueto J.A., Falcón J. (2008). Cloning and retinal expression of melatonin receptors in the European sea bass, *Dicentrarchus labrax*. Gen. Comp. Endocrinol.

[102] Mazurais D., Brierley I., Anglade I., Drew J., Randall C., Bromage N., Michel D., Kah O., Williams L.M. (1999). Central melatonin receptors in the rainbow trout: Comparative distribution of ligand binding and gene expression. J. Comp. Neurol.

[103] Gaildrat P., Falcon J. (2000). Melatonin receptors in the pituitary of a teleost fish: mRNA expression, 2-[(125)I]iodomelatonin binding and cyclic AMP response. Neuroendocrinology.

[104] Park Y.J., Park J.G., Hiyakawa N., Lee Y.D., Kim S.J., Takemura A. (2007). Diurnal and circadian regulation of a melatonin receptor, MT1, in the golden rabbitfish, *Siganus guttatus*. Gen. Comp. Endocrinol.

[105] Park Y.J., Park J.G., Jeong H.B., Takeuchi Y., Kim S.J., Lee Y.D., Takemura A. (2007). Expression of the melatonin receptor Mel(1c) in neural tissues of the reef fish *Siganus guttatus*. Comp. Biochem. Physiol. A Mol. Integr. Physiol.

[106] Iigo M., Furukawa K., Tabata M., Aida K. (2003). Circadian variations of melatonin binding sites in the goldfish brain. Neurosci. Lett.

[107] Amano M., Iigo M., Ikuta K., Kitamura S., Yamamori K. (2003). Characterization and maturational differences of melatonin binding sites in the masu salmon brain. Gen. Comp. Endocrinol.

[108] Gaildrat P., Ron B., Falcón J. (1998). Daily and circadian variations in 2-[125I]-iodomelatonin binding sites in the pike brain (*Esox lucius*). J. Neuroendocrinol.

[109] Planas J., Gutiérrez J., Fernández J., Carrillo M., Canals P. (1990). Annual and daily variations of plasma cortisol in sea bass *Dicentrarchus labrax* L. Aquaculture.

[110] Calduch-Giner J., Duval H., Chesnel F., Boeuf G., Pérez-Sánchez J., Boujard D. (2001). Fish growth hormone receptor: Molecular characterization of two membrane-anchored forms. Endocrinology.

[111] Bhandari R.K., Taniyama S., Kitahashi T., Ando H., Yamauchi K., Zohar Y., Ueda H., Urano A. (2003). Seasonal changes of responses to gonadotropin-releasing hormone analog in expression of growth hormone/prolactin/somatolactin genes in the pituitary of masu salmon. Gen. Comp. Endocrinol.

[112] Maestroni G.J., Conti A., Pierpaoli W. (1986). Role of the pineal gland in immunity. Circadian synthesis and release of melatonin modulates the antibody response and antagonizes the immunosuppressive effect of corticosterone. J. Neuroimmunol.

[113] Handy R.D. (2003). Chronic effects of copper exposure versus endocrine toxicity: Two sides of the same toxicological process?. Comp. Biochem. Physiol. A Mol. Integr. Physiol.

[114] Campbell H.A., Handy R.D., Sims D.W. (2002). Increased metabolic cost of swimming and consequent alterations to circadian activity in rainbow trout exposed to dietary copper. Can. J. Fish. Aquat. Sci.

[115] Fenwick J.C. (1970). Brain serotonin and swimming activity in goldfish, *Carassius auratus*. Comp. Biochem. Physiol.

[116] Nelson R.J. (1995). Biological Rhythms and Behaviour.

[117] Limson J., Nyokong T., Daya S. (1998). The interaction of melatonin and its precursors with aluminium, cadmium, copper, iron, lead, and zinc: An adsorptive voltammetric study. J. Pineal Res.

[118] Djeridane Y., Touitou Y. (2001). Melatonin synthesis in the rat harderian gland: Age- and time-related effects. Exp. Eye Res.

